# Evaluation of Liposome Encapsulated Propolis Nanoparticles on Cell Proliferation and Apoptosis in A375 Melanoma Cancer Cell Line

**DOI:** 10.1002/fsn3.70303

**Published:** 2025-05-23

**Authors:** Shima Bidabad, Hossein Ahmadpour Yazdi, Leila Zolghadr, Nassim Valivand, Nematollah Gheibi

**Affiliations:** ^1^ Department of Medical Biotechnology, School of Advanced Technologies in Medicine Qazvin University of Medical Sciences Qazvin Iran; ^2^ Cellular and Molecular Research Center, Research Institute for Prevention of Non‐Communicable Diseases Qazvin University of Medical Sciences Qazvin Iran; ^3^ Department of Chemistry Faculty of Science, Imam Khomeini International University Qazvin Iran; ^4^ Department of Chemistry Qazvin Islamic Azad University Qazvin Iran

**Keywords:** apoptosis, liposome, melanoma cancer cell, nanopropolis, propolis

## Abstract

Malignant melanoma is the deadliest type of skin cancer, and its global incidence has increased in the last decades. Recent studies have shown that propolis has an antitumor effect against various types of cancers. The aim of this study was to investigate the effects of Qazvin propolis nanoparticles encapsulated in liposomes on the A375 and HDF cell lines. For this purpose, the thin film hydration method was used to encapsulate nanopropolis within the liposomal formulation. Then, the physicochemical properties of the prepared liposomes were determined using DLS, FTIR, and SEM. In addition, the effects of this formulation on cell apoptosis, cell adhesion, cancer cell migration, and *BAX*, *Bcl‐2*, and *Caspase‐3* gene expressions were evaluated using flow cytometry, atomic force microscopy, scratch, and q‐real time PCR, respectively. According to the results, propolis nanoparticle‐liposomes have a cytotoxic effect on the A375 cell line in a dose‐ and time‐dependent manner through the induction of apoptosis, without having a toxic effect on the HDF cell line. The drug release results showed that more than 75% of the drug was released after 48 h at pH 5.4. The AFM and scratch analyses showed that Young's modulus and adhesion force values were increased. Therefore, this formulation significantly decreased the expression of *Bcl‐2* and increased the expression of *BAX* and *Caspase‐3* genes.

## Graphical abstract

Melanoma is the most aggressive type of skin cancer with an increasing rate in the last decades. Plant nano extracts such as nanopropolis are recommended for cancer treatment with fewer side effects and better effects. Liposomes containing plant compounds have been widely considered as targeted drug delivery systems in cancer treatment.

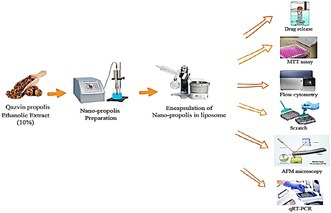

## 1 Introduction

Cancer is the second‐leading cause of death worldwide (World Health Organization 2022), and melanoma is the most aggressive type of skin cancer with an increasing rate in the last decades (Eriksson et al. 2015). This malignant tumor has a high metastasis rate and poor prognosis with a low survival rate of the patients (Goodson and Grossman 2009). The conventional treatment approaches for melanoma include surgery, chemotherapy, immunotherapy, and radiotherapy (Taher et al. 2023). Surgery is a common and effective treatment for early‐stage melanoma; however, if the tumors spread to other areas of the body, the treatment becomes more challenging, and the mortality rate increases (Davis et al. 2019; Naik 2021). Chemotherapy and radiotherapy are used in many types of cancers; however, they can lead to significant side effects such as lack of efficacy, damage to healthy cells, and drug resistance, which leads to failure in drug response (Naik 2021; Chapman et al. 2011).

Many studies have shown that natural products can reduce these adverse effects. Therefore, finding a natural product that is more efficient and less toxic is essential in cancer treatment. Propolis is a resinous natural substance that honeybees, namely *apis mellifera* , collect from cracks, barks, and leaf buds of plants (Forma and Bryś 2021). Propolis has a variety of therapeutic activities such as antitumor, anti‐inflammatory, antimicrobial, antioxidant, and anesthetic (El‐Guendouz et al. 2019). The chemical composition of propolis varies depending on the plant species, bee species, the location of propolis collection, and weather conditions; however, it mainly contains plant resins, waxes, essential oils, pollen, aromatic oils, and other organic compounds (Jayakumar et al. 2013; Chuttong et al. 2023). Recent studies have reported that propolis can influence key processes in the development of cancer including cell proliferation, angiogenesis, inhibition of apoptosis, and metastasis (Forma and Bryś 2021). Although propolis has various therapeutic activities, its resinous nature, low bioavailability, poor solubility, and physical instability limit the use of this compound (Trusheva et al. 2011). Due to the limitation of using free propolis, the use of Nanocarriers as a drug delivery system is a promising strategy. Various types of natural or synthetic nanocarriers have been used to treat melanoma cancer such as carbon nanotubes, gold nanoparticles, liposomes, niosomes, micelles, polymeric nanoparticles, and dendrimers (Chen et al. 2013).

Among all nanocarriers mentioned, liposomes have been widely studied as targeted drug delivery systems in cancer treatment (Mirzavi et al. 2021). Liposomes are spherical lipid‐based vesicles composed of phospholipid bilayers that surround an aqueous core. Liposomes are useful for targeted drug delivery because they can localize the drug to the site of action, which reduces the concentration of the drug in other parts of the body (Akbarzadeh et al. 2013). Liposomes represent a wide range of advantages including biocompatibility, biodegradability, entrapping both hydrophobic and hydrophilic drugs, low toxicity, high encapsulation capacity, and controlled drug release (Irie et al. 2003).

Apoptosis serves as the primary mechanism of cell death through which the organism seeks to remove transformed cells that may pose a cancer risk. Melanoma progression, similar to other cancer types, is influenced not only by changes in the genetic landscape, but also by a variety of molecular modifications in genes that regulate cell death, cell survival, and therapeutic responses. The ability to evade apoptosis is a defining characteristic of melanoma, playing a vital role in its progression, metastasis, and therapeutic resistance. In apoptosis regulation, the prosurvival members of the *Bcl‐2* protein family are frequently overexpressed in melanoma, contributing to the cancer's survival and its resistance to treatment (Trisciuoglio and Del Bufalo 2021). Additionally, apoptosis is the predominant form of cell death triggered by decarbonize and other agents that alkylate DNA. The process of DNA alkylation activates the intrinsic apoptosis pathway, a phenomenon that has been experimentally demonstrated in multiple melanoma model systems (Guttà et al. 2020).

In apoptosis, mitochondria are involved not only in caspase‐dependent apoptosis but also play a significant role in influencing the *Bcl*_*‐2*_ pathway during caspase‐independent apoptosis. Changes in mitochondrial structure are a key regulatory pathway for *Bcl* family proteins and caspase‐independent apoptosis. Among the *Bcl* family, *Bcl*_*‐2*_ and *Bax* are prominent proteins that either inhibit or promote apoptosis, respectively, and are essential in modulating mitochondrial membrane permeability, mitochondrial function, and the release of cytochrome. *Bcl*_*‐2*_ is predominantly found in the membranes of the nucleus, mitochondria, and endoplasmic reticulum, whereas proapoptotic members like *Bax* are primarily located in the cytoplasm (Wang et al. 2016).

Caspases represent a class of proteolytic enzymes that are integral to various cellular processes, particularly in the regulation of cellular mechanisms. They are essential for maintaining cell homeostasis and programmed cell death, known as apoptosis, as well as modulating nonapoptotic forms of cell death. The improper regulation of caspase activation is implicated in the development of several cancers, including melanoma. These enzymes are capable of both initiating and executing apoptosis, while also playing a critical role in the regulation of cell death and the management of tumor proliferation. Additionally, caspases contribute to the suppression of tumor growth by cleaving and deactivating proteins associated with cell proliferation and angiogenesis. Furthermore, they are involved in the activation of immune responses through the processing and presentation of tumor antigens, thereby enhancing the immune system's ability to recognize tumors. The involvement of caspases in melanoma is multifaceted, as they may serve to inhibit the growth and progression of this type of cancer (Szmurło et al. 2024).

Numerous studies have demonstrated that propolis can stimulate the apoptosis pathway by reducing the expression of the *Bcl*_*‐2*_ gene while enhancing the expression of the *BAX* gene. The regulation of the expression ratio between these genes is a critical factor that determines whether cells move toward or away from the apoptosis pathway. Consequently, the examination of *BAX* and *Bcl*_*‐2*_ gene expression serves as a significant method for evaluating the apoptosis pathway (Azarshinfam et al. 2021).

The present study aimed to prepare and design PEGylated liposomes loaded with propolis nanoparticles. The effects of this formulation on cell viability, apoptosis induction, inhibition of metastasis, and the expression of *BAX*, *Bcl*_*2*_, and *Caspase‐3* genes in cell line A375 were investigated. For this aim, MTT assay, flow cytometry, atomic force microscopy (AFM), scratch, and qRT‐PCR techniques were employed.

## 2 Materials and Methods

### 2.1 Material

Propolis was obtained from the Tarem region of Qazvin province, and an alcoholic extract was prepared from it. MTT was acquired from Sigma‐Aldrich, and additional consumables, including chloroform, were provided by Merck. Liposome synthesis lipids (HSPC, cholesterol, DSPE‐PEG2000) and a dialysis membrane were procured from Avanti (USA). DMEM High‐glucose was procured from Capricorn (Germany). The Annexin V‐FITC Apoptosis Kit was supplied by Invitrogen (Germany). The total RNA extraction kit and cDNA synthesis kit were purchased from YTA Co. (Iran) and SYBR Green qRT‐PCR Master Mix was procured from Ampliqon (Denmark).

#### 2.1.1 Preparation of Propolis Ethanolic Extract

Initially, to prepare a 30% ethanolic extract of propolis, Qazvin raw propolis was dissolved in 70% Ethanol for 2 weeks (200 g propolis in 1070 mL 70% ethanol) with agitation. Then, the propolis extraction was passed through Whatman filter paper three times to remove the impurities, and then it was placed on a rotary evaporator (Raha Tajhiz, Iran) to separate its organic solvent. After that, the propolis wax was separated and poured into a Petri dish and placed in a freezer at −20°C. Then, the solution was frozen, followed by lyophilization.

#### 2.1.2 Preparation of Nanopropolis

For preparing 8 mL nanopropolis, 0.24 g of freeze‐dried ethanolic extract of propolis was dissolved in 2 mL 80% ethanol. Then, tween 20 and tween 80 with a volume ratio of 1/2 were mixed homogeneously. The sonication of Propolis suspension with an amplitude of 30 W (On = 3 s and off = 8 s) was performed. Tween 20 and 80, distilled water, and propolis suspension were mixed together in 35 parts and sonicated. After that, the device was set to ON = 6 s and OFF = 4 s for 5 min, then OFF for 5 min, and this process was repeated again (王 et al. 2012). In addition, the following steps were performed to confirm the correctness of the process.

First test: 200 *λ* of propolis nanoparticles was centrifuged at 4000 rpm for 10 min. After that, no layer or no pellet was expected to form. Second test: 100 *λ* of nanoparticles was mixed with 100 *λ* of distilled water. It should be completely mixed, and no layer should be formed. Third test: A drop of nanoparticle was placed on the hemocytometer slide and observed under a light microscope; no large particle should be seen (王 et al. 2012).

#### 2.1.3 Propolis Encapsulated in Liposome Preparation

To prepare the nanopropolis encapsulated in liposomes, the thin film hydration method was employed. First, hydrogenated soy phosphatidylcholine (HSPC), cholesterol, and DSPE‐PEG2000 in a molar ratio of 70%:25%:5% were dissolved separately in the least volume of chloroform. The organic solvent was evaporated at 45°C under low pressure at 60 rpm using a rotary evaporator (Raha Tajhiz, Iran) until a dry thin film of nanopropolis lipid mixture was formed on the wall of the rotating container. The container was kept in the refrigerator for 24 h to remove the organic solvent. After that, the formed films were hydrated using an appropriate amount of phosphate buffer saline (PBS). The rotary flask was kept rotating in a water bath at ambient pressure at 45°C for 30 min until the film was completely hydrated. Then, the formed liposomes were kept at room temperature at 37°C to strengthen the liposome structure. During sonication, to prevent the temperature increase, the liposome suspension was placed into a cold‐water bath. A pulsed duty cycle was fixed for all treatments at 45 s ON and 15 s OFF intervals over 30 min. After sonication, the liposome suspension was filtered seven times using sterile MCE filters with a pore size of 0.22 μm (Sigma‐Aldrich, USA) (Odeh et al. 2012; El‐aziz et al. 2021). For the separation of encapsulated and unencapsulated nanopropolis, Amicon Ultra‐3 filter (3 kDa cutoff, Millipore, Germany) was used. For this process, the obtained suspension was centrifuged (5000 rcf, 15°C) for 30 min. Then the unentrapped and encapsulated nanopropolis was separated and stored at 4°C (Lopez‐Pinto et al. 2005).

#### 2.1.4 Evaluation of Nanopropolis Embedding Percentage (EE%)

After separating the entrapped and unentrapped nanopropolis, the amount of unencapsulated nanopropolis was determined by a spectrophotometer at a wavelength of 420 nm. The amount of free nanopropolis in the suspension was determined using a standard curve. The entrapment efficiency (EE%) was determined according to the below equation (Oreopoulou et al. 2019):

$$\mathrm{EE}\%=\frac{\text{Total amount of Nano propolis added}-\text{unentrapped Nano propolis}}{\text{Total amount of Nano propolis added}}\times 100$$

#### 2.1.5 Evaluation of Particle Size and Zeta Potential

To evaluate the average particle size, polydispersity index (PDI), and zeta potential of propolis nanoparticle, nanopropolis encapsulated in liposomes and empty liposomes and dynamic light‐scattering instrument (DLS) (Zetasizer ZS, Malvern Instruments, Worcestershire, UK) were applied with triplicate repetition (New 1990; Ruozi et al. 2005). The nanopropolis was diluted with ethanol 80%, and to avoid multiple light‐scattering effects, the liposome suspension was diluted with phosphate buffered saline. In this study, the stability of liposome containing nanopropolis was investigated in terms of size change and drug entrapment up to 14 days and 2 months (Sabaghi et al. 2024), and to get a correct insight into the stability, evaluation was also done after 7 months.

#### 2.1.6 FTIR

Fourier transform infrared spectroscopy (FTIR) (IRTracer‐100, Shimadzu) from 4000 to 400 cm^2^ was used to determine the quality of free nanopropolis, encapsulated nanopropolis, and free liposome samples. After taking the spectrum of functional groups of propolis, liposome, and liposome containing propolis were compared.

#### 2.1.7 Scanning Electron Microscopy (SEM)

For analyzing the surface characteristics and morphology of the liposome, scanning electron microscopy (SEM) (VEGA3, TESCAN) was used. The freeze‐dried nanopropolis‐liposome samples were coated with a gold film to prevent surface charging by the electron beam.

#### 2.1.8 Evaluation of Nanopropolis Release

For investigating the release percentage of nanopropolis from prepared liposomes at different pH, the dialysis membrane method was used. In brief, 1 mL of prepared liposomes was put in a dialysis bag (10 kDa) and placed into 5 mL of PBS at three pH levels: 7.4, 6.8, and 5.4. (Yang et al. 2007). The system was placed in a shaking incubator with continuous shaking at 300 rpm at 37°C. After dialysis started, 1 mL of samples was taken at specific times (0.5, 1, 2, 3, 4, 5, 6, 24, and 48 h) and replaced with the same amount of fresh medium. Then, the free drug concentration was measured using UV spectrophotometry at *λ*_max_ of 420 nm. The experiments were repeated three times.

### 2.2 Cell Culture

This study used human melanoma cancer cell line (A375) from the Iranian Biological Resource Center (IBRC) and Human Dermal Fibroblast (HDF) from Pasteur Institute (Tehran, Iran). The HDF cell line was used as a control to evaluate the cytotoxicity effect of this formulation. A375 and HDF cells were cultured in complete DMEM high‐glucose medium supplemented with 10% fetal bovine serum (FBS) and 1% penicillin/streptomycin and incubated at 37°C with 95% humidity and 5% CO_2_ in an incubator.

### 2.3 Cell Culture and Cytotoxicity Assay (MTT Assay)

To investigate the cytotoxic effect of liposomal nanopropolis on the desired cell lines, the MTT assay was performed. The MTT assay is the most common method that indicates metabolic activity and cell viability of cultured cells by converting the yellow MTT salt to purple formazan crystals, which determines the presence of living cells and mitochondrial activity (Shahab‐Navaei and Asoodeh 2023). For this purpose, A375 and HDF cells were cultured at a density of 5 × 10^3^ cells/well in 96‐well microplates. Following a 24 h incubation, the cells were treated with varying concentrations of liposomal nanopropolis for 24 and 48 h at 10, 20, 40,60,80, and 100 μg/mL (untreated cells and dimethyl sulfoxide (DMSO) 15% were used as a negative and positive control, respectively). After 24 and 48 h incubation, the medium was replaced with 100 μL of MTT solution (0.5 mg/mL) to each well and incubated for 4 h in the CO_2_ incubator. MTT reagent is light‐sensitive because exposure can lead to the degradation of formazan crystals; therefore, this procedure should be conducted in the dark. Then, the MTT solution was discarded, and 200 μL of dimethyl sulfoxide (DMSO) was placed in the wells and incubated for 15 min to dissolve formazan salt crystals. Finally, the absorbance was measured at 570 and 630 nm through microplate readers (Biotech Instruments, USA) (El‐aziz et al. 2021; CD Creative Diagnostics n.d.; Vafaei et al. 2020). The experiments were done in three repetitions, and the results were analyzed and checked using the following formula:

$$\%\text{Cell Viability}=\frac{\text{Control sample}-\text{treated sample}}{\text{Control Sample}}\times 100$$

#### 2.3.1 Analysis of Apoptosis Induction

To investigate the apoptosis effect of nanopropolis loaded liposome on the A375 cell line, Flow cytometry technique was used. Flow cytometry is a commonly method that enables the detection and quantification of apoptosis rate as well as the percentage of necrosis within a population of treated cells. In this technique, a double staining approach was employed utilizing FITC‐Annexin V to identify exposed phosphatidylserine, which translocate from the inner cytoplasmic membrane to the cell surface in early apoptotic cells, alongside propidium iodide (PI), which binds to and labels DNA fragments in late apoptotic cells (Shabani et al. 2020). Briefly, the A375 cells (25 × 10^4^) were seeded in six‐well plates for 24 h. After that, the cells were treated with IC_50_ 24 and 48 h of nanopropolis loaded liposome incubated for 24 and 48 h at 37°C. After harvesting the cells, Further steps were performed according to the kit protocol (Invitrogen kit). In other words, all harvested cells were centrifuged at 1500 rpm for 5 min. Then, 300 μL of binding buffer (1×) was added to the cell pellet after washing with cold PBS. Then, 2 μL of FITC‐Annexin was added to the samples and incubated for 15 min in the dark. After that, 1 μL of propidium iodide (PI) were added to the samples. Finally, the samples were detected by the flow cytometry device (BD Biosciences, San Jose, CA, USA). The analysis of flow cytometry results was conducted utilizing Flowjo software (Zolghadr et al. 2023).

#### 2.3.2 Atomic Force Microscopy (AFM)

Melanoma cells (25 × 10^4^) were seeded in Petri dishes and placed in a CO_2_ incubator at 37°C for 24 h. After that, the A375 cells were treated with nanopropolis‐liposome (44 mg/mL, 24 h) and (20 μg/mL, 48 h) at 37°C. For cell fixation process, the cells were fixed by glutaraldehyde solution (0.5%) for 1 min after washing with PBS. Then, glutaraldehyde solution was removed. In the last step, after removing the PBS, the cells were dried at room temperature. After preparation of the cells, to evaluate the nanomechanical, morphological properties and elastic behavior of treated cells, Nanowizard‐2 AFM was performed in noncontact mode at T:37 ± 2°C (Petri Dish Heater, JPK Instrument). A sensitive V‐shaped tip with 10 nm radius, side angle 10° and nominal spring constant 0.07–0.35 N/m was installed on the stand. Moreover, for quality control of images standardization methods such as ISO 5725, ISO/IEC 17,042, and ASTM E691 were used. This equation was also employed for calculating the young's modulus:

$$F=\frac{2}{\pi }\tan\left(\alpha \right)\frac{E}{1-v^{2}}\delta^{2}$$

*F* = loading force, *E* = Young's modulus, *v* = Poisson ratio at 0.5 is suitable for cells, *δ* = Indentation, *α* = Half of a conical tips opening angle (Pi et al. 2016).

#### 2.3.3 Scratch–Wound Assay

Initially, melanoma cells (3 × 10^5^) were seeded on six‐well plates and incubated at 37°C in a CO_2_ incubator for 24 h. After that, when the cell confluence reached 90%, a thin straight line was created with a sterile pipette tip in each well. After washing the cells with PBS, the cells were treated with IC_50_ 24 h and IC_50_ 48 h concentrations of nanopropolis loaded in liposomes. The cell migration into the wound space was observed and captured using an inverted microscope (CK53 model from Olympus, Japan) at 0, 4, 8, 12, 24, and 48 h and compared with the control. For data analysis, AutoCAD software was used. First, the leading edges of the wound position were detected; then, the migration rate was quantified by measuring the wound closure over the time mentioned.

#### 2.3.4 Evaluation of Gene Expression

To evaluate the efficacy of nanopropolis‐liposome on the expression of the *BAX*, *Bcl*_*‐2*_, and *Caspase‐3* genes, the qRT‐PCR was performed. First, A375 cells (3 × 10^5^) were seeded on six‐well plates and incubated at 37°C for 24 h. Then, the cells were treated with a nanopropolis‐liposome (44 mg/mL, 24 h). After 24 h, RNA was extracted from harvested cells according to the total RNA extraction kit protocol. Subsequently, the complementary DNA (cDNA) synthesis was performed on extracted total RNA using the GeneAll kit. The qRT‐PCR SYBR green master mix (Amplicon, Denmark), and Rotor Gene real‐time PCR machine 6000 (Qiagen, USA) were used to carry out real‐time PCR. As an internal control, the *GAPDH* gene was used, and SinaColon (SinaColon Co., Iran) was used to synthesize primer sequences. Finally, to analyze relative gene expression data (*BAX, Bcl*_*‐2*_, and *Caspase‐3*), the 2^−ΔΔCt^ comparative method was employed. The sequence of the primers used for qRT‐PCR and cyclin conditions for *BAX*, *Bcl*_*‐2*_, and *Caspase‐3* genes are shown in Tables 1, 2, 3 respectively.

**TABLE 1:** Sequences of the primer pairs used for qRT‐PCR.

Gene	Reverse	Forward
*GAPDH*	5′‐TGGAAGATGGTGATGGGATT‐3′	5′‐CAATGACCCCTTCATTGACC‐3′
*BAX*	5′‐CTCAGCCCATCTTCTTCC‐3′	5′‐GCCTCCTCTCCTACTTTG‐3′
Bcl_‐2_	5′‐GCATTCTTGGACGAGGG‐3′	5′‐TGGGAAGTTTCAAATCAGC‐3′
*Caspase‐3*	5′‐ACCGAGCTCCGAGGGCGGGAG‐3′	5′‐GCGGTAGCGCCGTCCGTTGC‐3′

**TABLE 2:** Cyclin conditions of *BAX* and *Bcl*_*‐2*_ genes for qRT‐PCR.

Stage	Cycle	Temperature (°C)	Time
Initial Denaturation	1 cycle	95	5 min
Stage 1 (Denature)	30 cycle	95	40 s
Stage 2 (Annealing)		57	40 s
Stage 3 (Extend)		72	40 ss

**TABLE 3:** Cyclin conditions of *Caspase‐3* gene for qRT‐PCR.

Stage	Cycle	Temperature (°C)	Time
Initial denaturation	1 cycle	95	**7** min
Stage1 (Denature)	40 cycle	95	30 s
Stage2 (Annealing)		56	30 s
Stage3 (Extend)		72	20 s

### 2.4 Statistical Analysis

The data were analyzed using one‐way ANOVA. The calculation of meaningful differences between data groups was performed using Duncan's multiple range test in SPSS software version 20 (SPSS, Armonk, NY, USA). For AFM data analysis, the *t*‐test, *F*‐test, and *Z*‐scores were applied in SPSS and JPK software. All graphs in this article were drawn using GraphPad Prism8 software (*p* < 0.05).

## 3 Results

### 3.1 Liposome Morphology Examination

**FIGURE 1:**
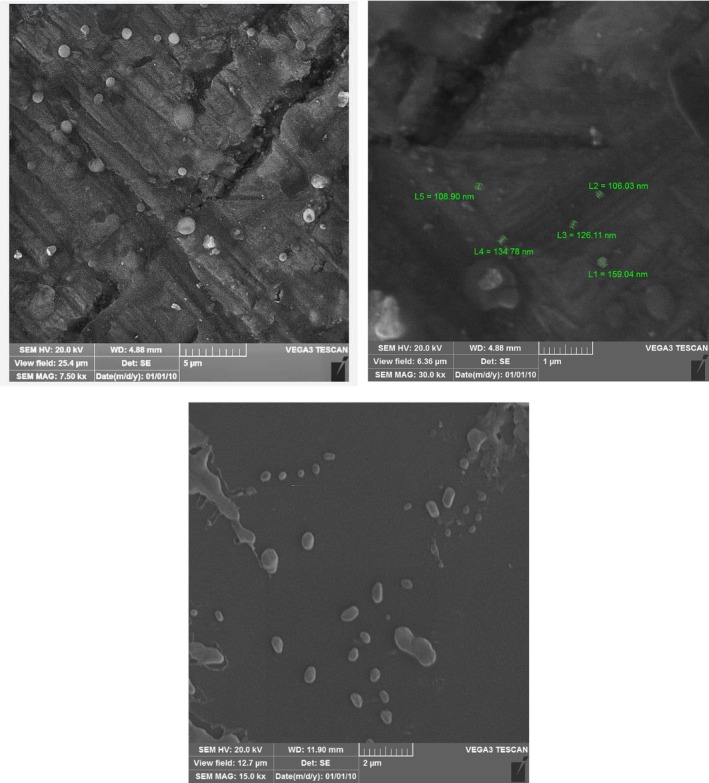
SEM images of (A, B) nanopropolis‐liposome, and (C) empty liposomes.

Scanning electron microscopy (SEM) was used to examine the morphology, shapes, and size distribution of empty and nanopropolis entrapped in liposomes. SEM images confirmed that prepared liposomes had an integrated and spherical structure and were well distributed with a homogenous size of nanometer dimensions (Figure 1).

### 3.2 Characterization of Free Nanopropolis and Nanopropolis‐Liposome

The size, the surface charge, and the homogeneity of the synthesized nanoparticles are important factors in determining the stability, the biological behavior, and their half‐life in the blood circulation as determined by DLS, zeta potential, and PDI, respectively. The results of encapsulation efficiency (EE%), DLS, zeta potential, and multiple dispersion index (PDI) of nanopropolis, nanopropolis‐liposome, and free liposome on the Zero‐time, fourteenth day, and 3 months after encapsulation are shown in Tables 4 and 5. As you can see in Table 5, there is an increase in particle size after 2 months, which may be attributed to the tendency of some vesicles to stick together. However, the variation in size is still acceptable and shows stability in the formulated liposome.

**TABLE 4:** Characterization of free nanopropolis, nanopropolis‐liposome, and empty liposome.

	Size (nm)	Zeta potential (mV)	PDI	EE%
Nanopropolis	3.97	−14.02	0. 21	—
Nanopropolis‐liposome	186.2	−14.8	0.17	—
Free liposome	95.74	−3.8	0.11	84.21%

**TABLE 5:** Stability study of nanopropolis‐liposome after 14 days and 2 months of storage at 25°C.

Formula (nanopropolis‐liposome)	Zero‐Time	After 14 days	After 2 months
Size (nm)	186.2	189.8	201
EE%	84.21%	76.3%	65.1%

### 3.3 Fourier Transform Infrared Spectroscopy (FTIR)

**FIGURE 2:**
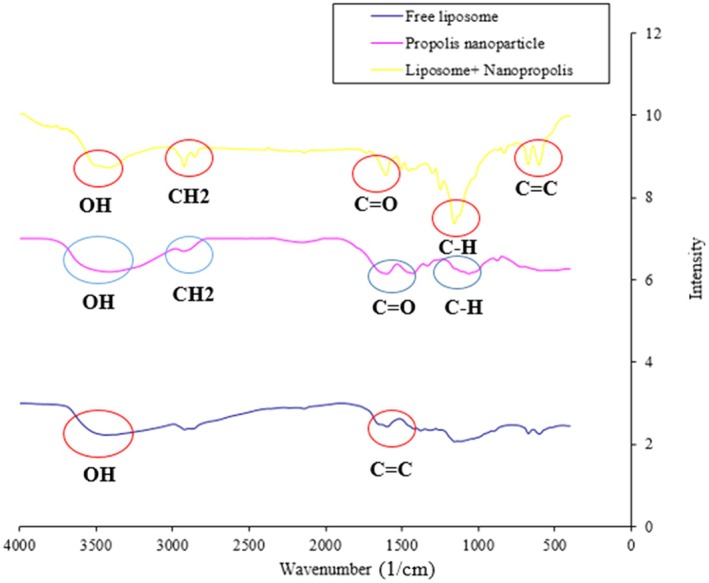
FTIR spectra of free nanopropolis, nanopropolis‐liposome, and empty liposome.

The potential chemical interaction between nanopropolis‐liposome, free liposome, and nanopropolis was evaluated by the FTIR analysis (Figure 2). The FTIR spectrum obtained from the empty liposome showed the absorption band at 80–100 and 1600 cm^−1^ that indicated the presence of an aromatic ring and the CC stretching, respectively. Moreover, the bands observed at 3320 and 3471 cm^−1^ indicated the –OH bonds stretching. Additionally, the absorption band in the 2500–2900 and 3300–3700 cm^−1^ regions were associated with the C‐H and OH functional groups, respectively. In the FTIR spectrum of nanopropolis, the absorption at 693, 812, and 984 cm^−1^ corresponds to the C=C group. In addition, the bands at 1259 and 1692 cm^−1^ were associated with C=O, and the peak at 1036 cm^−1^ represented the S=O groups. Additionally, the absorption bands at 1110, 1446, 2855, and 3332 cm^−1^ were associated with C‐O, CH, CH_2_, and OH groups, respectively. The FTIR spectrum of liposomal nanopropolis showed 2 sharp peaks at 627 and 947 cm^−1^ that indicated the presence of the C=C group. The peaks observed at 1383–1460, 1736, 2867, and 3700 cm − ^1^ are ascribed to C‐H, C=O, CH_2_, and OH, respectively, which indicates the penetration of nanopropolis into the prepared liposomes.

### 3.4 In Vitro Release

**FIGURE 3:**
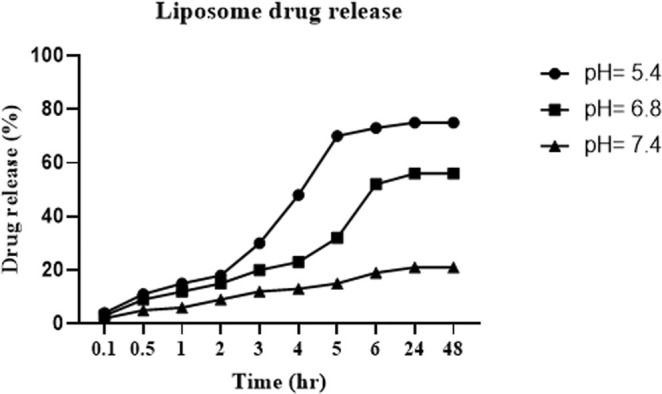
Release of nanopropolis from the prepared liposomes in time intervals (0.5‐48 h) at 3 pH: 5.4, 6.8, and 7.4.

The release of nanopropolis from liposomes at pH levels of 7.4 (physiological), 6.8 (tumor environment), and 5.4 (endosomal environment) was evaluated over a 48‐h period. As shown in Figure 3, the highest amount of nanopropolis release occurred within 48 h. Additionally, the release increased with the decreasing pH of the environment. Approximately 75% of the nanopropolis was released at pH 5.4 after 48 h, confirming the enhanced performance of nanopropolis in the tumor environment.

### 3.5 Cell Viability Assay

**FIGURE 4:**
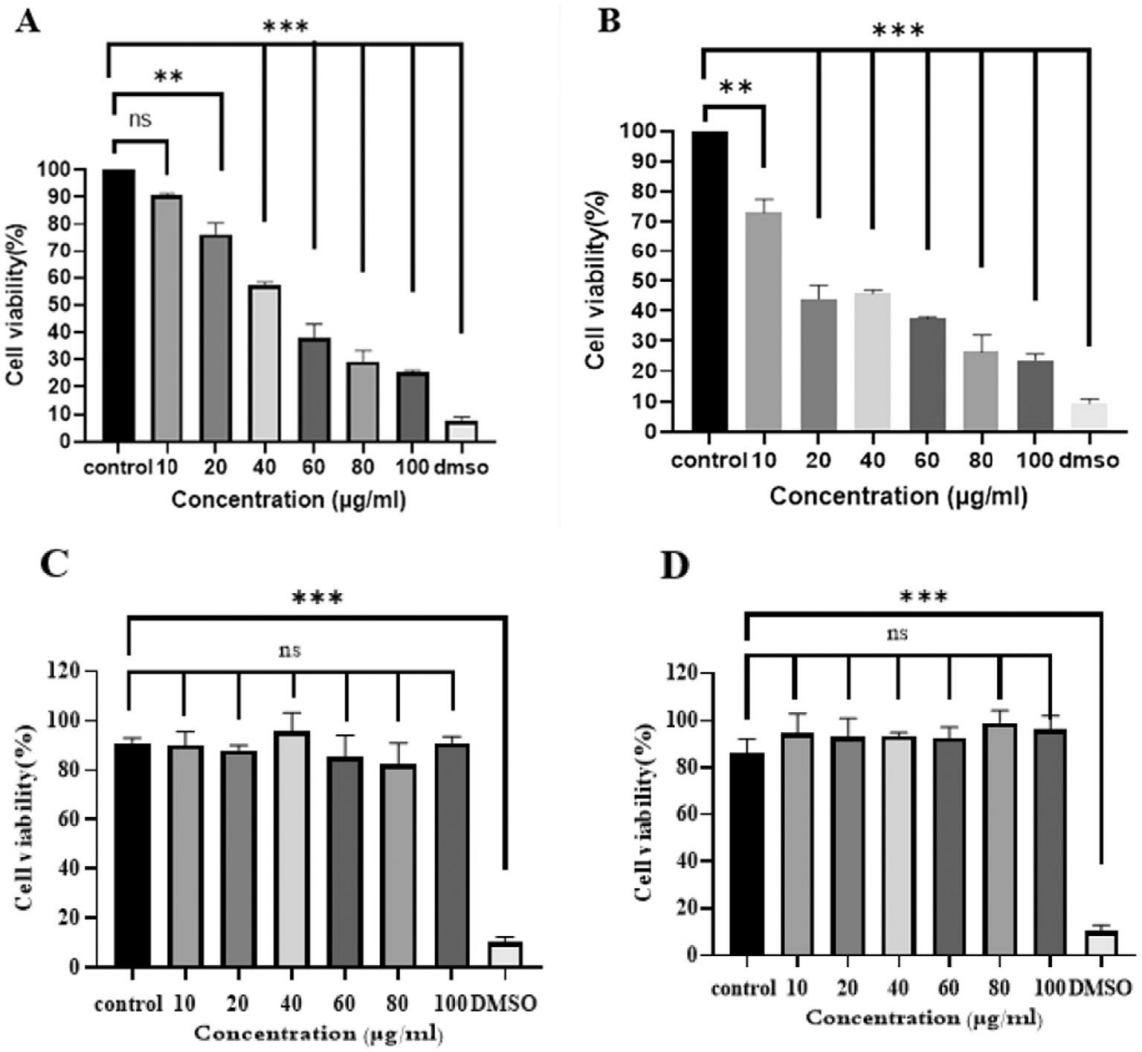
Toxicological results of nanopropolis‐liposome on the A375 for (A) 24 h and (B) 48 h and on the HDF for (C) 24 h and (D) 48 h. (*p* value < 0.01**/*p* value < 0.001***).

The cell viability of A375 and HDF cell lines after being treated with increasing concentrations of nanopropolis encapsulated in liposomes for 24 and 48 h was investigated through MTT assay. The 24 h incubation studies showed that nanopropolis‐liposome at lower concentration (10 μg/mL) did not show significant toxicity; whereas, with increasing concentrations, the cell viability was reduced significantly. According to the result of 48 h incubation, the cell viability was further reduced, indicating a higher extent of cytotoxicity. Eventually, the IC_50_ values of treated cells were calculated as 44 and 20 μg/mL for 24 and 48 h, respectively. In addition, the HDF cell line was employed to assess the potential cytotoxic effects of liposomal nanopropolis on normal cells. The results of the MTT assay indicated that different concentrations of liposomal propolis nanoparticles exhibited no cytotoxic effects on the HDF cell line. Furthermore, after 48 h, this formulation demonstrated a proliferative effect on the normal cell line. Finally, these results showed that nanopropolis‐liposome decreased cell viability in a dose‐ and time‐dependent manner on the A375 cell line and confirmed its anticancer effects (Figure 4).

### 3.6 Flow Cytometry

**FIGURE 5:**
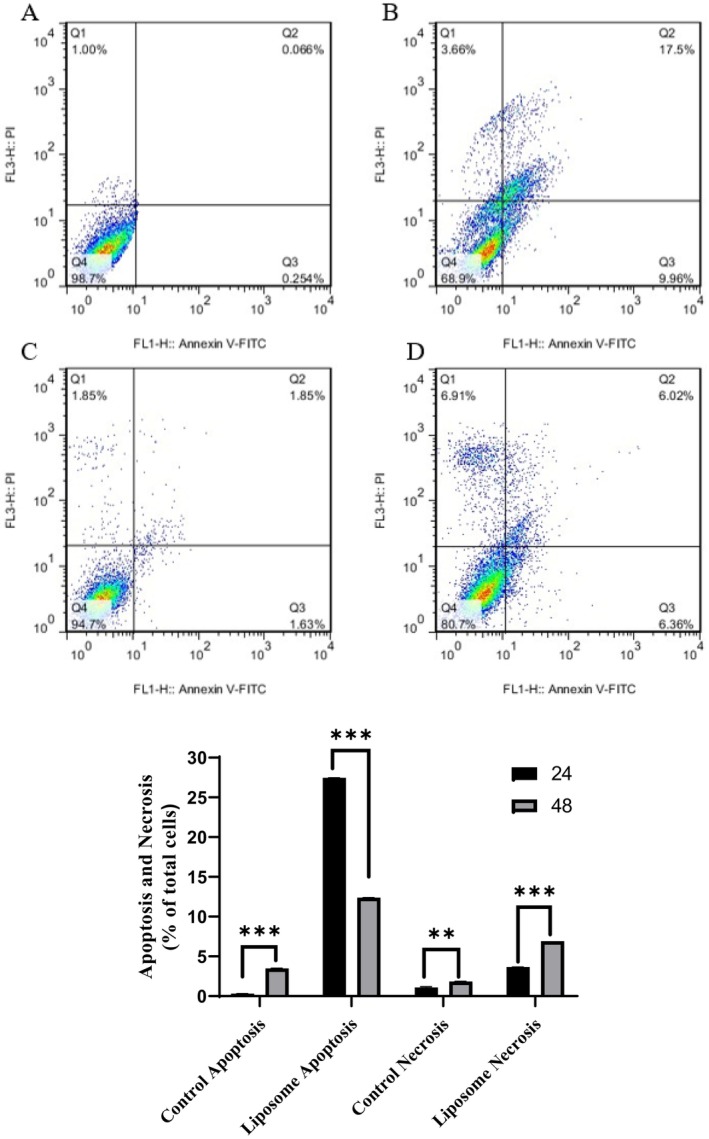
Proapoptotic effects of nanopropolis‐liposome in the A375, (A) untreated cells after 24 h, (B) A375 cells treated with IC50 24 h, (C) untreated cells after 48 h. (D) A375 cells treated with IC_50_ 48 h. (*p* value < 0.01**/< 0.001***).

The A375 cells were treated with IC_50_ 24 for 48 h (44 and 20 μg/mL) to investigate its proapoptotic effects through Annexin V/PI. The distribution of cell populations was categorized into four distinct areas: normal (Q4), necrosis (Q1), early apoptosis (Q3), and late apoptosis (Q2). Based on the results of flow cytometry, the total apoptotic percentage (early apoptosis + late apoptosis) of the treated cells after 24 and 48 h was 27.46% and 12.38%, respectively, and the percentage of necrosis cells was 3.66% and 6.91%, respectively. These results confirmed that nanopropolis‐liposome had a great cytotoxic effect on the A375 cell line and its apoptosis induction was more significant after 24 h compared to 48 h (Figure 5).

### 3.7 Mechanical Properties

**FIGURE 6:**
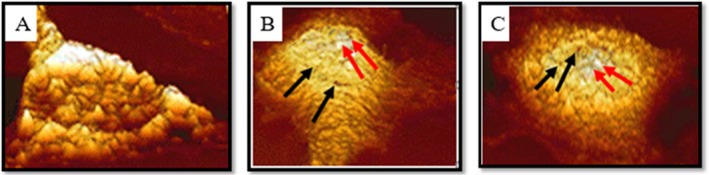
Structural changes in A‐375 cells and the formation of apoptotic cavities after the treatment of cells. (A) untreated cells, (B) A375 cells treated with IC50 24 h, (C) A375 cells treated with IC50 48 h.

The results showed nanomechanical and morphological changes after treatment. According to the results, treatment of cells with nanopropolis‐liposomes triggered changes in the structure of the cell cytoplasmic membrane compared with the control group. The white and brown pores, indicating apoptotic and necrotic bodies around the cytoplasmic membrane, are observed in treated cells in Figure 6. There was a direct relationship between the values of Young's modulus and the cell–cell adhesion force (Lu et al. 2020) (Table 6). The treated cells with 20 μg/mL nanopropolis‐liposome (IC50 48 h) had more cell–cell adhesion force compared with the 44 μg/mL concentration (IC_50_ 24 h). These results indicated that nanopropolis‐liposome can prevent the invasion and metastasis of the A375 melanoma cancer cell line.

**TABLE 6:** Nanomechanical changes in A‐375 cells after treatment with liposome‐nanopropolis.

Compounds	Mean young's modulus value (kPa) ± SE	Mean adh. force (pN) ± SE	Mean Z pulling (μm) ± SE
Control	1.78 ± 0.01	1.6 ± 0.02	1.56 ± 0.1
Nanopropolis+ liposome after 24 h	3.011 ± 0.03	1.81 ± 0.05	1.72 ± 0.025
Nanopropolis+ liposome after 48 h	4.32 ± 0.05	2.12 ± 0.07	2.85 ± 0.08

### 3.8 Scratch–Wound Assay

**FIGURE 7:**
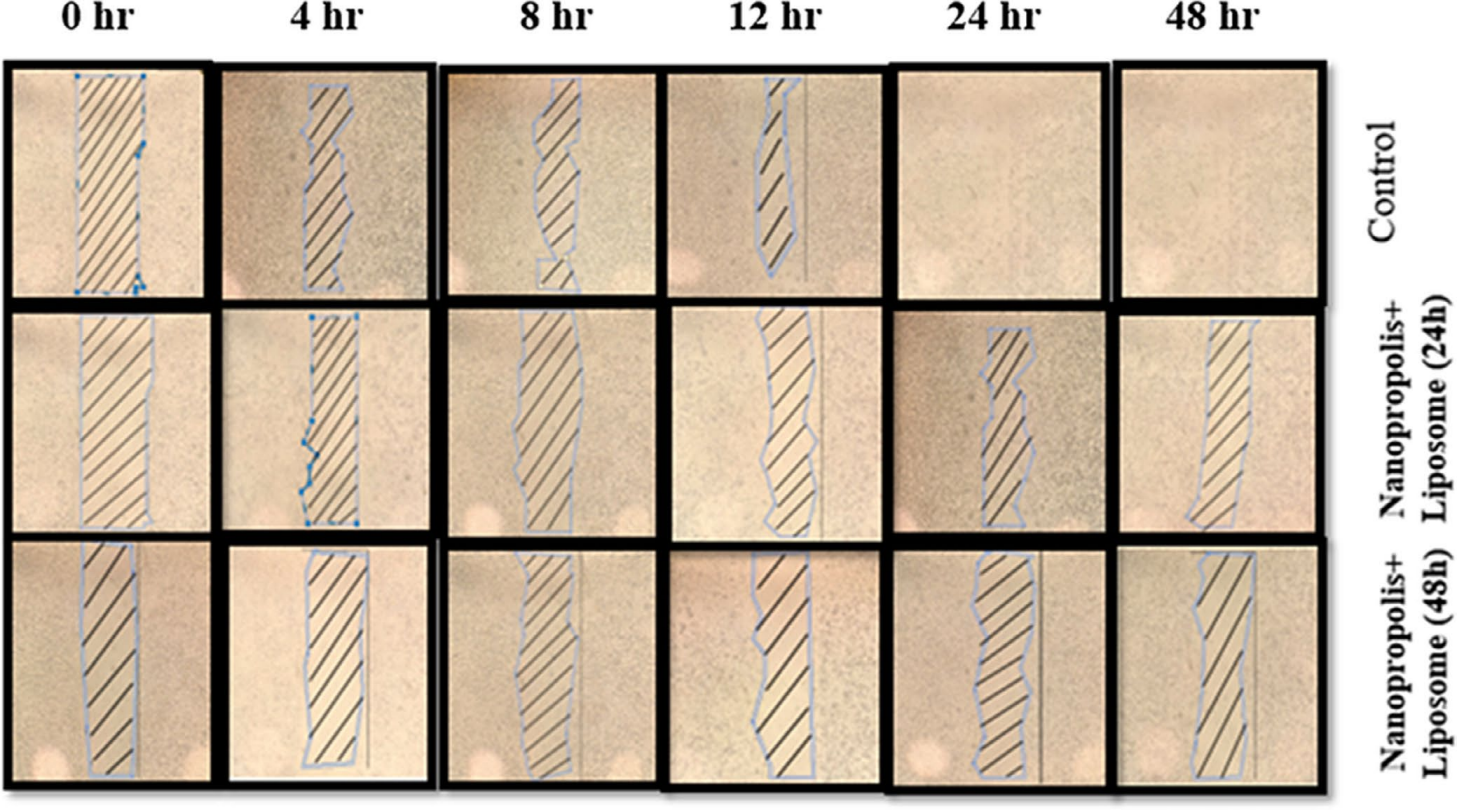
Scratch assay showing the inhibition of migration of treated cells with IC_50_ 24 and 48 h.

The assay was performed to investigate the effects of nanopropolis‐liposome on the migration of A375 cells. Based on the results, nanopropolis‐liposome significantly inhibited the treated cell's migration in a time‐dependent manner. As shown in Figure 7, scratch–wound width was suppressed at both IC_50_ concentrations, and the number of cells that migrated to the scratch site was significantly lower compared to untreated cells. In addition, the results showed that cells treated with IC_50_ 48 h can inhibit the migration of melanoma cells more than 24 h.

### 3.9 Gene Expression Study

**FIGURE 8:**
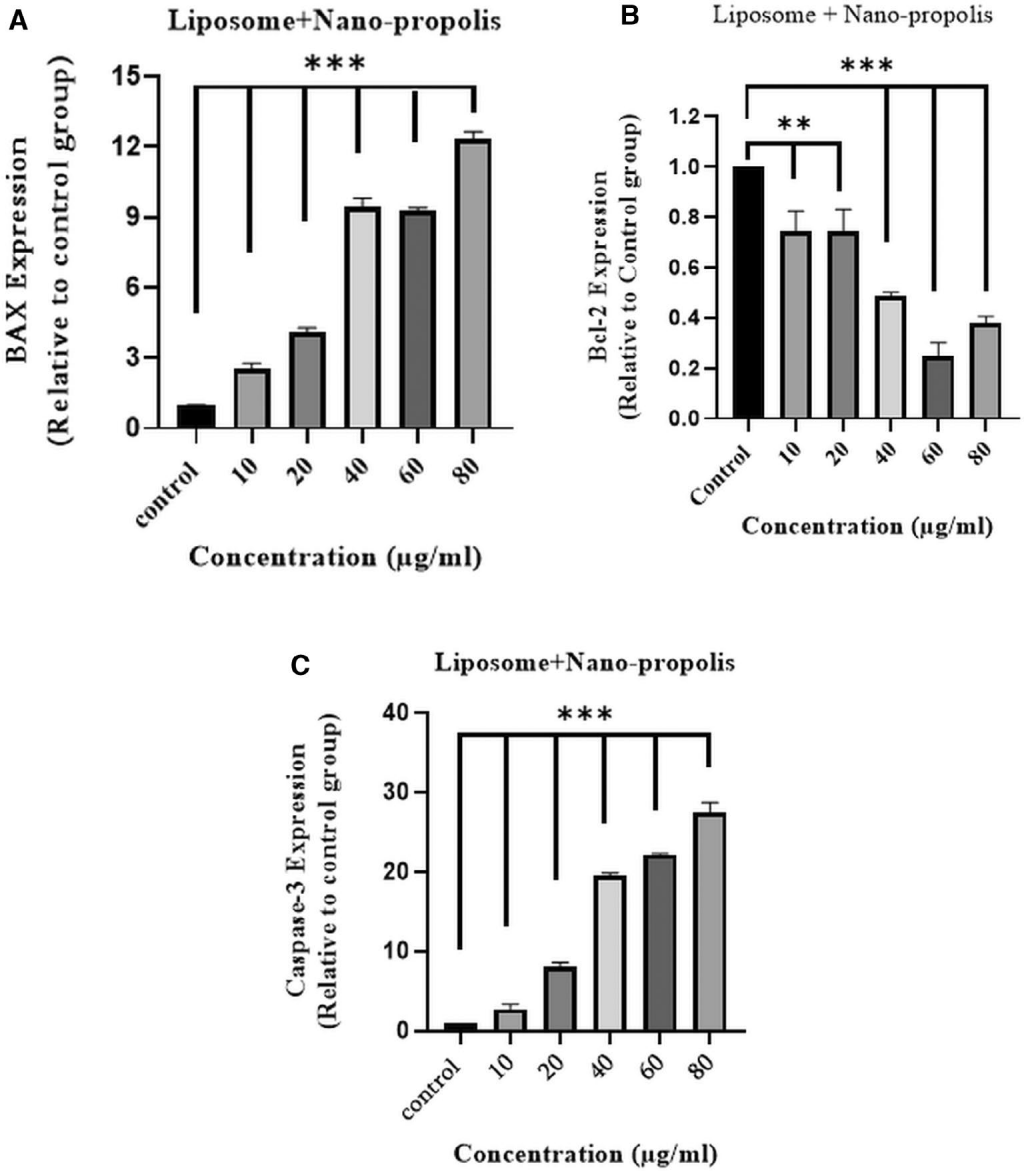
Level expression of *BAX, Bcl‐2*, and *Caspase‐3* using real time PCR method. A375 cells were treated with different concentrations of liposome‐nanopropolis for 24 h. Compared with untreated cells, (A, C) the expression of *BAX* and *Caspase‐3* increased and, (B) the expression of *Bcl‐2* decreased. (*p* value < 0.01**/< 0.001***).

In this study, the qRT‐PCR was used to investigate the proapoptotic activity of the nanopropolis‐liposome against the A375 cell line. As shown in Figure 8 nanopropolis‐liposome affects the expression of *BAX*, *Bcl*_*‐2*_, and *Caspase‐3* genes compared with control. These results demonstrated that by increasing the concentration of liposome‐nanopropolis, the gene expression of *caspase‐3* and *BAX* also increased, while the expression of antiapoptotic *Bcl*_*‐2*_ decreased.

## 4 Discussion

Natural products have great benefits in the treatment of diseases. Among the various compounds, propolis is one of the strongest natural products with antiulcer, antidiabetic, anti‐inflammatory, and antitumor properties. Although propolis has various useful therapeutic activities, its use is limited due to its instability, low water solubility, and resinous nature. Nowadays, nanotechnology has emerged as a new promising approach for the diagnosis and treatment of various types of cancers. Among all carriers used in nanotechnology, liposomes are the most successful for drug delivery to the target cell. So, in this research, we encapsulated nanopropolis in nano‐sized liposomes to overcome these limitations. Liposome properties vary depending on lipid composition, surface charge, size, and method of preparation (Akbarzadeh et al. 2013). For liposome synthesis, hydrogenated soy phosphatidylcholine (HSPC), cholesterol, and DSPE‐PEG2000 were employed. HSPC has a relatively high phase transition temperature that increases the stability of the liposome structure. In addition, an increase in the percentage of HSPC concentration caused the formation of more liposome vesicles (Pezeshky et al. 2016). Studies have shown that an increase in cholesterol percentage increases encapsulation efficiency (EE%), which may be due to the enhanced stability of the prepared liposomes. As cholesterol increases in the lipid layer of liposomes, the rigidity and stability of the lipid bilayer increase. However, by decreasing the cholesterol content, the size of liposomes decreases, which needs a smaller nanocarrier and enhances the cellular uptake of the entrapped drug (El‐aziz et al. 2021). In addition, DSPE‐PEG 2000 was used in the liposomal formulation. By conjugating DSPE‐PEG into the lipid membrane, the clearance of liposomes by the reticuloendothelial system was limited and resulted in prolonged liposome circulation (Gaumet et al. 2008). Therefore, 70:25:5 ratios of HSPC, cholesterol, and DSPE‐PEG2000 were used for liposome synthesis.

One of the most important factors in the liposome synthesis is the size, which affects their penetration capability and circulation time in the body. Nanocarriers should be synthesized in small particle size to avoid clearance in the reticuloendothelial system and mononuclear phagocytic recognition (Ramli et al. 2021). Li et al. (2016) showed that liposomes with a size range of 100–300 nm had an excellent ability to deliver bioactive agents to target cells. However, larger liposomes (larger than 500 nm) are more likely to clear rapidly before reaching the bloodstream (Ramli et al. 2021). Several studies have demonstrated the relationship between zeta potential and liposome stability (Makino et al. 1991).

In our study, liposomes had a desirable zeta potential with a negative surface charge. This negative surface charge can prevent nanoparticle aggregation by producing electrical repulsion and can reduce negative interactions between nanoparticles and plasma proteins and/or red blood cells (Suk et al. 2016). Also, the results of the stability study of the formulated liposome showed that after 2 months the size of the particles is increasing, but the stability is acceptable. The release percentage of nanopropolis from prepared liposomes was evaluated at three different pH levels (5.4, 6.8 and 7.4) for 48 h under similar conditions. The results showed that the release rate depended on pH, and by decreasing the environmental pH, the release percentage was increased. Thus, the highest drug release rate was in acidic pH (pH = 5.4). Aytekin et al. (2020) showed that encapsulated propolis into liposomes exhibited a controlled release tendency at pH: 7.2 for 8 h. This formulation could release the maximum amount of entrapped drug in the tumor's surrounding environment compared with the healthy tissues, which causes healthy tissue to be less exposed to the drug.

To investigate the effects of encapsulated nanopropolis on melanoma cancer cell line (A375) and human dermal fibroblast (HDF), an MTT assay was used. As mentioned before, in this method, the serial dilution of nanopropolis‐liposome was prepared, and the cell viability of treated cells was evaluated for 24 and 48 h. According to the results, this liposome formulation decreased the viability of treated cells in a time and dose‐dependent manner, and the viability of the treated cells was lower after 48 h compared to 24 h (IC50 24 h 44 μg/mL and IC50 48 h 20 μg/mL). According to Refaat et al. (2019), encapsulated propolis into liposomes led to inhibition of the growth of A375 compared to the unencapsulated propolis in a dose‐dependent manner. In addition, no toxicity was observed on human fibroblasts, and after 48 h, a proliferation and healing effect was also observed, which means that this formulation can be suggested for wound healing. Additional studies seem to be effective. However, the MTT assay cannot distinguish whether the loss of cell viability is due to apoptosis or necrosis; therefore, other techniques were used to determine these differences. To determine the molecular mechanisms involved in the cytotoxic activity of nanopropolis‐liposome, Flow cytometry was performed. Annexin V not only distinguishes apoptotic cells from necrotic cells but also binds to phosphatidylserine on the surface of apoptotic cells to determine whether apoptotic cells are in the early or late apoptotic phase (Vermes et al. 1995). Our results indicated that nanopropolis‐liposome induced early and late apoptosis in treated cells; however, the rate of induction of total apoptosis in treated cells with IC_50_ 24 h was found to be higher than that in those treated with IC50 48 h. Flow cytometry results of El‐aziz et al. (2021) indicated that propolis encapsulated in liposome induced apoptosis of Hep‐2 cell line, and the effect was greater compared to propolis extract.

AFM is used to study the mechanical and dynamic properties of intact cells associated with many cellular events, including aging, migration, differentiation, cell pathology and activation, and electrical change. AFM can perform surface imaging and ultrastructural observations of living cells with atomic resolution close to physical conditions, gathering powerful spectral information to study the mechanical properties of cells. In this study, the cells were examined with atomic force microscopy and the Young's modulus and cell–cell adhesion were found to be the highest in cells treated with the nanopropolis‐liposome. Similar to our results, the inverse relationship between Young's modulus and malignancy has been confirmed in previous studies. Because malignant cells are less adherent and more flexible, this may be due to changes in actin fiber organization and changes in cytoplasmic pressure and density (Zolghadr et al. 2023; Deng et al. 2018). The migration and invasion ability of A‐375 cells were examined by scratching cancer cells and it was found that liposome‐nanopropolis treatment delayed the recovery of scratched melanoma cancer cells. The results showed that when cells were treated with nanopropolis‐liposome, the number of cells migrating to the scratch site was also lower compared to untreated cells. This indicates that the nanopropolis‐liposome can significantly inhibit the migration of A375 cells. According to Xuan et al. (2014), ethanolic extract of Chinese propolis can significantly inhibit the migration of MDA‐MB‐231 breast cancer cell lines in a dose‐dependent manner after 48 h. Another study showed that Brazilian red propolis ethanolic extract inhibited the migration of 5637 Bladder Cancer Cells significantly after 24 h (Begnini et al. 2014). Apoptosis is mediated by two main pathways: the extrinsic or death pathway and the intrinsic or mitochondrial pathway. The intrinsic apoptotic pathway is mediated by mitochondria and regulates the balance and interaction between pro‐ and antiapoptotic members of the *Bcl*_*‐2*_ family of proteins, which control mitochondrial membrane permeability. Both pathways lead to the activation of *caspases*, which terminate cell survival. The *BAX* gene is a proapoptotic member of the *Bcl*_*‐2*_ gene family and plays an important role in the apoptotic pathway because it increases its expression, forms homo‐ or heterodimers with the *Bcl*_*2*_ protein and induces cell death (Begnini et al. 2014; Carpenter and Brady 2020). The study found a correlation between increased apoptosis induced by the liposomal form of nanopropolis and increased levels of *BAX* and *Caspase‐3* protein in treated cells, which heterodimerizes and inhibits *Bcl*_*‐2*_. Azarshinfam et al. (2021) indicated the effects of propolis in combination with double hydroxide nanoparticles on the expression of *BAX* and _*Bcl‐2*_. The results showed that LDH nanoparticles significantly increased the expression of the *BAX* gene and decreased the expression of the *Bcl*_*‐2*_ gene and induced apoptosis in HT‐29 cell lines compared to propolis extract (Azarshinfam et al. 2021). Another study showed that Portuguese Propolis increased the expression of apoptotic genes (*BAX*, *Caspase‐3*, *caspase‐9*, and *P53*) and decreased the expression of antiapoptotic genes (*Bcl‐*_*2*_ and *Bcl‐XL*) in the A375 melanoma cancer cell line (Oliveira et al. 2022). These results show that propolis can induce apoptosis in different cancer cell lines.

## 5 Conclusion

The nanopropolis encapsulated into liposomes has a cytotoxic effect and the ability to induce apoptosis in melanoma cancer cell lines (A375). Moreover, this compound reaches its maximum release at pH 5.5. In addition, the AFM results showed that Young's modulus increased in treated cells. Also, the high expression of *BAX* and *Caspase‐3* genes in the treated cells confirmed the induction of apoptosis by nanopropolis. These findings confirmed that Qazvin nanopropolis encapsulated in liposome has antitumor activity against the A375 cell line and that this formulation may be a promising candidate as a potential natural agent for development of an anticancer drug for melanoma cancer in the future. In the end, with the obtained results, nanopropolis extract can be suggested as an herbal composition for the treatment of skin disorders.

## Author Contributions

**Shima Bidabad:** data curation (equal), formal analysis (equal), methodology (equal), project administration (equal), software (equal), writing – original draft (equal). **Hossein Ahmadpour Yazdi:** investigation (equal), supervision (equal). **Leila Zolghadr:** data curation (equal), formal analysis (equal), software (equal), supervision (equal), validation (equal), visualization (equal), writing – original draft (equal). **Nassim Valivand:** data curation (equal). **Nematollah Gheibi:** conceptualization (equal), methodology (equal), supervision (equal), visualization (equal), writing – review and editing (equal).

## Conflicts of Interest

The authors declare no conflicts of interest.

## Data Availability

We confirm that all the data and findings of this study are available within the article.
